# Collagen supplementation and regenerative health: advances in biomarker detection and smart material integration

**DOI:** 10.3389/fnut.2025.1716166

**Published:** 2025-12-11

**Authors:** Tatjana Ivaskiene, Jonas Viskelis, Paulina Streimikyte, Milda Savickaitė, Ali Mobasheri, Greta Kaspute

**Affiliations:** 1 State Research Institute Centre for Innovative Medicine, Vilnius, Lithuania; 2 Lithuanian Research Centre for Agriculture and Forestry, Institute of Horticulture, Babtai, Lithuania; 3 Research Unit of Health Sciences and Technology, Faculty of Medicine, University of Oulu, Oulu, Finland; 4 Department of Joint Surgery, First Affiliated Hospital of Sun Yat-sen University, Guangzhou, Guangdong, China; 5 Faculty of Medicine, Université de Liège, Liège, Belgium

**Keywords:** collagen, supplement, clinical trial, biological effect, biosensors, drug delivery systems

## Abstract

Collagen, the most abundant structural protein in the human body, plays a key role in skin integrity, tissue repair, and extracellular matrix organization. With increasing consumer and clinical interest, collagen supplementation has expanded rapidly, yet scientific evidence supporting its efficacy in anti-aging and regenerative applications remains inconsistent. This review critically evaluates current evidence on oral collagen supplementation, integrating insights from over 60 clinical studies assessing its effects on skin aging, musculoskeletal health, and hair disorders. Emerging data suggest that hydrolyzed collagen peptides may improve skin elasticity, joint function, and recovery after exercise, particularly when co-supplemented with vitamin C, silica, or resveratrol. Beyond supplementation, recent advances in biosensing and material science have enabled novel approaches to collagen detection and targeted delivery. Electrochemical, optical, and molecularly imprinted polymer (MIP)-based biosensors facilitate real-time monitoring of collagen biomarkers such as CTX-I, MMPs, and hydroxyproline, supporting precision assessment of collagen metabolism. In parallel, collagen-based hydrogels, nanoparticles, and electroresponsive scaffolds have shown promise as biocompatible carriers for controlled drug release and regenerative therapy. Collectively, these developments outline a translational framework connecting collagen supplementation, detection, and delivery. Continued integration of biosensing and smart material technologies may enhance clinical monitoring and therapeutic efficacy, advancing collagen-based interventions toward precision dermatology and regenerative medicine.

## 1 Introduction

Collagen is the most abundant structural protein in the human body, comprising approximately 30% of total protein content (1). As a key component of connective tissues—including skin, cartilage, tendons, and bone—collagen plays a fundamental role in maintaining tissue architecture, mechanical strength, and cellular signaling. In the skin, particularly the dermis, collagen types I and III provide tensile strength and elasticity, both of which decline significantly with age due to intrinsic and extrinsic factors such as ultraviolet (UV) exposure, hormonal changes, and oxidative stress (2).

Growing interest in anti-aging and skin health has fueled the development of collagen-based interventions, particularly oral supplements and topical formulations containing hydrolyzed collagen peptides (CPs) (3). Preliminary studies suggest that CPs, especially those derived from bovine or marine sources, may improve skin hydration, elasticity, and dermal density (4). These effects are hypothesized to arise from enhanced fibroblast activity, increased synthesis of extracellular matrix proteins such as elastin, and reduced expression of matrix metalloproteinases (MMPs) that degrade native collagen. However, while *in vitro* and small-scale clinical studies offer promising results, robust evidence from large randomized controlled trials (RCTs) remains limited (5).

The rapid expansion of the collagen supplement market—valued at nearly USD 2 billion in 2021—has outpaced scientific consensus, with many products marketed as broad-spectrum solutions for aging skin, hair loss, and joint health (6). This discrepancy underscores the need for objective biomarkers to assess collagen metabolism and treatment efficacy (7). Emerging technologies, such as electrochemical sensors and molecularly imprinted polymers (MIP) (8), offer potential for non-invasive, sensitive, and selective detection of collagen or its metabolites in biological fluids (9–11). These tools could enhance both clinical research and personalized dermatologic care by enabling real-time monitoring of collagen-related interventions (12, 13).

This review aims to fill that gap by providing a comprehensive, interdisciplinary synthesis of current clinical evidence, detection technologies, and biomaterial innovations related to collagen. It highlights how biosensors and electroresponsive delivery systems can enhance monitoring, efficacy, and safety of collagen-based interventions. The target audience includes clinicians, biomedical researchers, and biomaterials scientists seeking to understand how advances in sensing and delivery can translate nutritional supplementation into clinically meaningful outcomes in dermatology, regenerative medicine, and anti-aging therapeutics. In addition, despite the growing number of publications on collagen supplementation and biomaterial applications, the field remains fragmented across nutrition, dermatology, and materials science. Previous reviews have primarily focused on either the biochemical properties of collagen or its cosmetic and orthopedic applications, without integrating emerging evidence from biosensing and advanced delivery systems. Consequently, there is a lack of a unified framework linking collagen supplementation, real-time detection technologies, and drug delivery platforms, which together could enable personalized and feedback-controlled collagen therapies.

## 2 Methods

**Figure 1:**
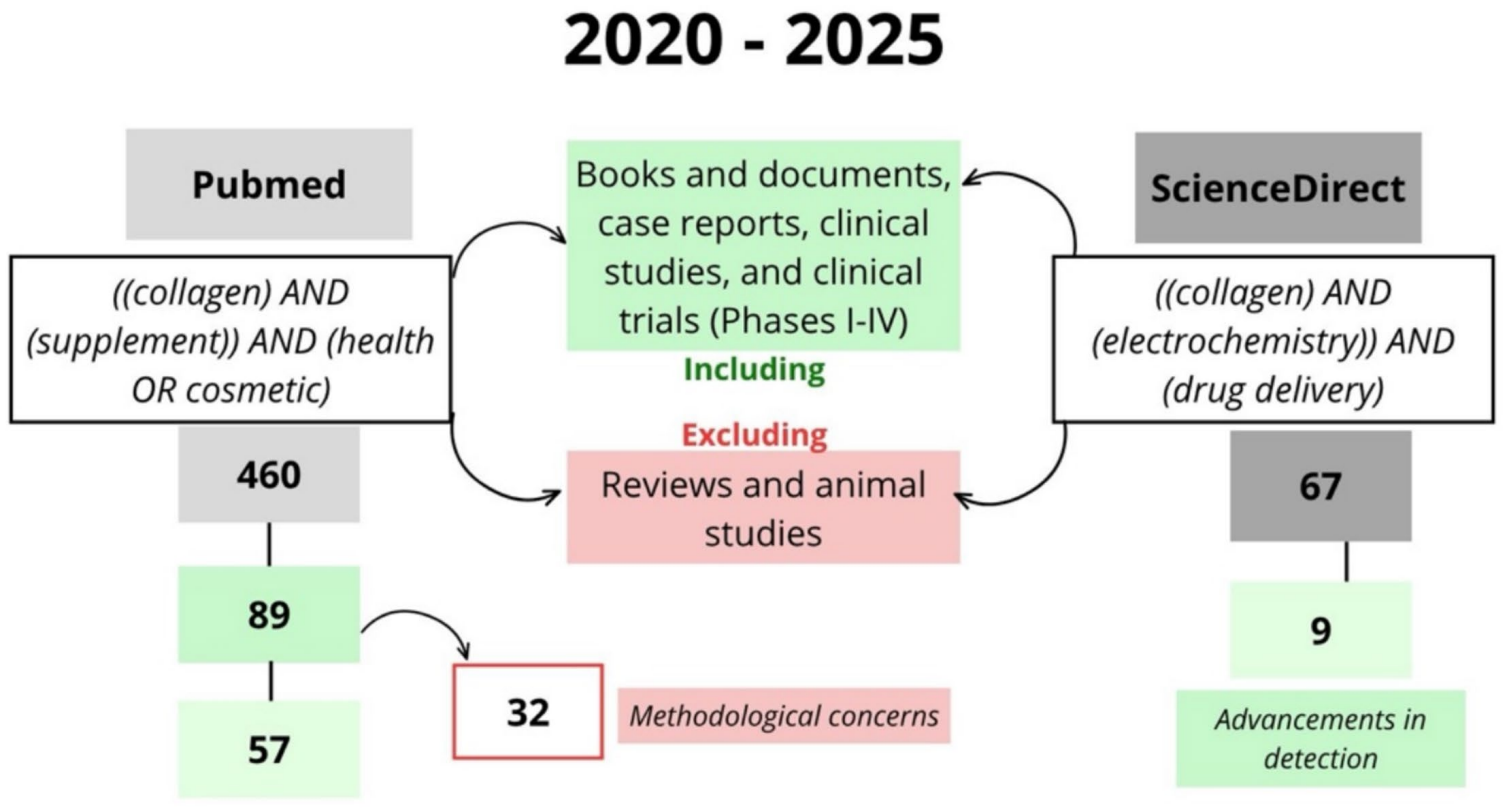
Schematic representation of the search strategy employed in this review article.

A comprehensive literature search was conducted using PubMed and ScienceDirect to identify studies investigating the effects of collagen supplements on health and cosmetic outcomes as well as electrochemical studies related to collagen (Figure 1).

### 2.1 Search strategy

The search was performed for publications from January 2020 and October 2025 to ensure inclusion of the most recent data. The following terms were used for this search:

1. Pubmed: ((collagen) AND (supplement)) AND (health OR cosmetic)
2. ScienceDirect: (collagen AND electrochemistry) OR (collagen AND drug delivery)

Filters were applied to include books and documents, case reports, clinical studies, and clinical trials (Phases I-IV), and exclude reviews and animal studies.

Studies were selected according to predefined criteria to ensure relevance and methodological rigor (Table 1).

**Table 1:** Inclusion and exclusion criteria.

Criteria	Inclusion	Exclusion
Publication period	2020–2025	Prior to 2020
Study type	RCTs, observational studies, meta-analyses, controlled clinical trials, validation studies, pragmatic clinical trials, and clinical trial protocols	Narrative reviews, editorials, conference abstracts
Population	Human participants of any age or sex	Animal or *in vitro* studies
Intervention	Collagen supplementation (oral, injectable, or topical) or collagen-based electrochemical/drug-delivery systems	Non-collagen-related interventions
Outcome	Health, cosmetic, or analytical/electrochemical outcomes related to collagen	Irrelevant or indirect outcomes

### 2.2 Screening and methodological assessment

Following the initial search, a total of 460 articles were identified, with 89 studies being deemed relevant for inclusion in the review. Of the eligible studies, 32 were excluded after further review due to methodological concerns, such as insufficient sample size or lack of control group, incomplete outcome reporting, non-standardized collagen formulations or dosages, or lack of statistical analysis. In the final, this process resulted in leaving 57 studies that met the inclusion criteria for analysis.

In addition, to compile relevant data for this review, a comprehensive search was conducted using ScienceDirect, focusing on peer-reviewed research articles. The search terms were “collagen,” “electrochemistry,” and “drug delivery.” A total of 67 articles were identified, of which 9 met the inclusion criteria following relevance screening. These studies focused on the role of collagen in biosensing and drug delivery systems, emphasizing electrochemical detection and monitoring strategies. The summarized schematic representation of the search results is presented in Figure 1.

Data from all included studies were extracted manually, summarizing author, year, study design, sample size, collagen source and dosage, duration and main findings. The results were synthesized narratively due to heterogeneity in study designs and outcomes.

## 3 Clinical evidence and biological effects of collagen supplementation

Collagen supplementation has emerged as a promising intervention in dermatologic and musculoskeletal health, supported by a growing body of clinical and molecular evidence. Recent RCTs demonstrate that bioactive CPs, particularly from marine and bovine origins, exert targeted effects on skin elasticity, hydration, wrinkle reduction, and joint function.

### 3.1 Molecular mechanisms of collagen action

Orally ingested hydrolyzed collagen is absorbed as di- and tripeptides (e.g., Pro-Hyp, Gly-Pro-Hyp) that act as signaling molecules to activate dermal fibroblasts, upregulate hyaluronic acid synthesis, and stimulate expression of collagen types I and III (14–17). These peptides also inhibit MMP-1 and MMP-3, reducing collagen degradation, particularly under UV or oxidative stress conditions (5, 14). *In vitro* studies further confirm that collagen peptides enhance elastin production and fibroblast proliferation, effects potentiated by co-supplementation with antioxidants, silicon, or vitamins D and E, (15–20) all of which support enzymatic hydroxylation of proline residues necessary for collagen stabilization. Silicon, in nanoparticle form, has been shown to directly upregulate collagen gene expression and enhance skin barrier function in human trials (21).

Collagen turnover in the skin is intricately regulated by mechanical tension, cytokine signaling, and cellular senescence (22). Mechanical forces exerted by dermal fibroblasts are essential for maintaining extracellular matrix (ECM) integrity. In young skin, fibroblasts adhere to a robust collagen network, generating tension that promotes collagen synthesis and suppresses MMP activity (23). However, with aging, collagen fibers become fragmented, leading to reduced mechanical tension, fibroblast collapse, and diminished collagen production. This loss of tension also downregulates transforming growth factor-beta (TGF-*β*) signaling by decreasing TGF-β type II receptor expression, further impairing ECM maintenance (24, 25). Reduced fibroblast contractility and chronic inflammation (inflammaging) contribute to ECM fragmentation and impaired repair (26). Targeting these mechanisms through functional foods, microbiome-derived bioactives [e.g., *L. plantarum* (27)], and polyphenols such as resveratrol and fermented pomegranate extract represents a new frontier in dermatologic anti-aging strategies (28, 29).

### 3.2 Clincal evidence and personalised effects of collagen

The reviewed studies collectively demonstrate encouraging evidence for collagen supplementation across dermatologic and systemic outcomes. However, methodological diversity, inconsistent dosing regimens, and variable outcome measures complicate direct comparisons. A critical appraisal of recent RCTs reveals both convergence in certain findings and notable gaps in standardization and mechanistic understanding. For example:

1. Skin hydration and elasticity (within 4–12 weeks) with 2.5–10 g/day of marine or porcine collagen peptides (30–32).
2. Wrinkle depth reduction, especially periorbital lines, in postmenopausal women (31).
3. Hair thickness and reduced shedding, when collagen was combined with micronutrients and antioxidants (33).
4. Nail brittleness, where improvements were observed after 6 months of supplementation (34).

A broader spectrum of clinical trials evaluating collagen supplementation across dermatologic and systemic indications is summarized in Table 2.

**Table 2:** Clinical trials on collagen supplementation.

Trial	Condition	Therapy	Results	Ref.
RCT	Obesity	The bovine collagen with enhanced water retention capacity. Collagen supplementation: *n* = 64, 20 g per day for 12 weeks.	↓body weight, body mass index (BMI), fat mass, hunger sensations ↑ fat-free mass	(42)
RCT	Pain	Naturagen® 4 Joint product containing type I, II, and III collagen. *N* = 31, 20 g/day for 8 weeks.	↑ pain, quality of life, kinesiophobia, and foot function, leg strength No significant changes were found in back strength, proprioception, or other functional tests.	(35)
		3 treatment groups of recreational athletes with early osteoarthritic changes in the knee: collagen, exercise, and collagen plus exercise. *N* = 48, collagen, exercise or both for 12 weeks.	All groups: ↓ pain, but the exercise therapy alone and combined with collagen enhanced knee strength and function. The combination of collagen and exercise: ↑ knee range of motion, strength compared to exercise alone.	(37)
		The intra-articular injections of atelocollagen on knee pain in patients with osteoarthritis and cartilage defects. Male C57BL/6 mice (8 weeks old, *n* = 5/group); ACLT-induced OA; intra-articular injection of PBS, geraniol (1 mg/mL), or geraniol@nanogel weekly × 8 weeks.	↓ knee pain, as measured by the Visual Analog Scale, compared to the placebo group at 24 weeks.	(36)
		The 12-week supplementation with 5 g of specific CPs in a diverse group of healthy adults with functional joint pain. *N* = 182, 5 g/day specific CPs for 12 weeks.	↓ pain at rest, while walking, climbing stairs, kneeling down, and squatting for those taking CPs compared to the placebo group.	(96)
		The CPs supplementation for 12 weeks. *N* = 167, 10 g/day CPs for 12 weeks.	↑ knee function in CPs and placebo groups. No significant differences between the groups in terms of pain reduction or changes in inflammatory, cartilage, or bone biomarkers.	(97)
Double-Blind, Placebo-RCT		A chicken HC type II collagen for adults aged 40–65. *N* = 90, 2.5 g/day of chicken HC type II collagen for 8 weeks.	↓ joint pain, stiffness ↑ mobility	(98)
Double-Blind RCT		The long-term effects of daily CPs supplementation (10 g/d and 20 g/d) in middle-aged active adults. *N* = 86, 10 g/day or 20 g/day of CPs for 3–9 months.	Over 6 months, 10 g/d of CPs: ↑ activities of daily living, mental health scores with 10 g/d over 3–9 months, physical health scores with 20 g/d, but only in females. ↓ pain in those exercising over 180 min per week. While.	(41)
Double-blind RCT		“Wellnex” Type J CPs as a nutritional supplement for knee joint osteoarthritis in comparison to conventional bovine CPs. 5 groups (*n* = 100) receiving various dosages of Type J CPs (2.5, 5.0, 10.0 g), conventional CPs (10.0 g), or placebo for 90 days.	Type J CPs (2.5 g, 5.0 g, and 10.0 g) were all found to be beneficial for treating knee OA, with 2.5 g of Type J being as effective as 10 g of conventional CPs.	(99)
Assessor-blinded RCT	Androgenic alopecia and chronic telogen effluvium	An oral supplement containing fish HC, taurine, cysteine, methionine, iron, and selenium for *n* = 83 for 12 weeks together with their drug treatment. Group A (*n* = 48): Oral supplementation (1 tablet/day) + standard drug treatment for hair loss (AGA/FAGA or TE) Group B (*n* = 35): Standard drug treatment only	↑ global assessment scores (GAS) at both week 6 and week 12 compared to those receiving drug treatment alone, with 50% of the supplement group achieving a GAS score of ≥2 versus 23% in the control group. The supplement was well tolerated, suggesting it effectively enhances hair loss treatment outcomes.	(33)
RCT	Post exercise recovery/ muscle strength	Dietary CPs in middle-aged males unfamiliar with exercise. *N* = 20, 10 g/day of CPs or placepo for 33 days.	↓ muscle soreness and fatigue immediately after exercise ↑ muscle strength 48 h later	(100)
		Specific CPs combined with resistance training (RT) on body composition and muscle strength in middle-aged, untrained men, with a comparison to whey protein supplementation. *N* = 97, Collagen peptides (CPs-G, 15 g/day, *n* = 30); Placebo (P-G, *n* = 31) or whey protein (WP-G, 15 g/day, *n* = 36) for 12 weeks.	Collagen peptide group had greater ↑ fat-free mass, ↓ fat mass compared to the placebo group. Whey protein group also saw positive changes, but not as pronounced as the collagen peptide group.	(101)
		The specific CPs combined with RT on tendinous and muscular properties in 40 healthy male volunteers. Over 14 weeks of high-load RT, one group received 5 g of CPs daily, while the other received a placebo.	CPs group: ↑ Achilles tendon cross-sectional area (CSA) (+11.0%) and muscle thickness (+7.3%) compared to the placebo group (+4.7% and +2.7%, respectively). While tendon stiffness and muscle strength increased in both groups, there were no significant differences between them.	(43)
		Comparison of HC and dairy protein (DP) effects on recovery from exercise-induced muscle damage in 33 males who consumed either CH, DP (25 g protein), or a placebo immediately post-exercise and daily for 3 days. Active males. Participants consumed the supplement drink 2 h before the post-exercise follow-up visits at 24, 48, and 72 h.	Downhill running significantly induced muscle soreness decreased muscle function, and increased biomarkers of muscle damage and inflammation. However, no differences were observed between the CH, DP, and placebo groups in any recovery outcomes.	(102)
		Daily supplementation with 40 mg of undenatured collagen for 96 participants over 35 years old for 24 weeks.	↑ knee range of motion flexion and extension in healthy individuals with activity-related joint discomfort.	(103)
		The dose–response effect of HC ingestion before resistance exercise (RE) on collagen turnover in middle-aged, resistance-trained men. 8 participants (average age: 49 ± 8 years) consumed 0 g, 15 g, or 30 g of vitamin C-enriched HC before performing lower-limb RE. Single session per intervention was performed. Each participant completed all 3 interventions,	The 30 g HC: ↑ significantly increased serum PINP concentration (a marker of collagen synthesis) compared to both 15 g and 0 g HC.	(44)
Placebo-RCT		The specific CPs supplementation combined with RT for 50 healthy, moderately active men completed a 14-week RT program (3 sessions per week at 70–85% of their 1-repetition maximum) for the knee extensors. The CP group received 5 g of specific collagen peptides daily, while the control group received a placebo.	↑ patellar tendon cross-sectional area compared to the placebo group, particularly at 60 and 70% of the tendon length from the proximal insertion. CP supplementation combined with RT: ↑ patellar tendon hypertrophy.	(104)
RCT	Mood, fatigue, and physical condition	CPs in healthy but easily fatigued individuals. *N* = 33, 10 g/day for 8 weeks.	Participants who consumed 10 g/day of CPs for 8 weeks: ↓ perceived fatigue ↑ vigor, compared to the placebo group. The active group: ↑ feeling more rested after sleep, though no significant differences were found in immunological parameters.	(38)
		Combination of jump training with collagen supplementation on BMD in elite road-race cyclists. The 18-week intervention included *n* = 36, five 5-min bouts of jumping exercise per week, preceded by 15 g of HC.	Results showed that BMD at the femoral neck was preserved in the intervention group, while it decreased in the control group.	(40)
Open-Label RCT	Skin	Daily oral supplementation with CPs (10 g) on skin hydration and elasticity in 39 adults aged 65 and older for 2, 4, 6 and 8 weeks.	↑ stratum corneum hydration, skin elasticity compared to baseline.	(32)
Triple-Blined, Placebo-RCT		Marine HC (Vinh Wellness Collagen, VWC), 10 g/day dissolved in ≥100 mL water, consumed in the morning on an empty stomach for *n* = 50, 12 weeks.	↓ 35% in wrinkle scores, ↑skin elasticity, particularly in women aged 45–54.	(31)
RCT		Oral supplementation with CPs rich in X-Hyp or X-Hyp-Gly. *N* = 30, 5 g/day of collagen for 6 weeks.	↑ skin elasticity and collagen synthesis compared to normal collagen hydrolysates.	(30)
		A 12-week daily supplementation with 1,650 mg of CPs, *n* = 100.	↑ skin hydration, desquamation, wrinkling, and elasticity in women aged 30 to 60, with noticeable effects as early as 4 weeks.	(47)
		A 16-week daily supplementation with HC and vitamin C. *n* = 90, CPHA group: 5 g collagen + 30 mg hyaluronic acid + 80 mg vitamin C/day (15 mL syrup); CPs group: 5 g collagen + 80 mg vitamin C/day (15 mL syrup); Placebo group: 15 mL syrup without active ingredients. Duration: 16 weeks.	↑ dermis density, skin texture, ↓ wrinkle severity in women aged 40–65.	(105)
		Daily supplementation with 5 g of CPs for 84 days in East 85 asian women aged 43–65.	↑ dermis density, skin moisture, elasticity, wrinkle visibility, nail color, and beauty perception were observed within 28 days in the collagen group, while similar effects took 84 days in the placebo group.	(34)
Double-Blind, Placebo-RCT		Water-soluble coenzyme Q10 (Q10Vital®) and collagen in 34 healthy women aged 40–65, for 12 weeks. Test group: 10 mL/day syrup containing hydrolysed fish collagen 4 g, CoQ10 50 mg, vitamin C 80 mg, vitamin A 920 μg, biotin 150 μg; Placebo group: 10 mL/day syrup without active ingredients.	↑ dermis density, skin smoothness. ↓ periorbital wrinkle area, total wrinkle score.	(45)
RCT	Sleep	Glycine-rich CPs supplementation. *N* = 13, 15 g/day of CPs 1 h before bedtime for 7 nights.	↓ nighttime awakenings ↑ improved cognitive performance the following morning in physically active men with sleep complaints.	(106)
Crossover RCT	Gastrointestinal (GI) stress	20 volunteers (16 males) participated in 3 trials: a non-exercise rest trial (REST), and two exercise trials with either CPs supplementation (10 g/day) or a placebo control. The supplementation was consumed for 7 days prior to and 45 min before a 70-min run at 70–90% VO_2_max.	No significant differences between the CPs and placebo trials in heart rate, perceived exertion, thermal comfort, core temperature, or GI symptoms during exercise.	(107)

Some explanations of the used abbreviations and symbols: ↓ - reduction, ↑ - increase/improvement, RC – randomized controlled trial.

Beyond dermatology, collagen supplementation also improved musculoskeletal parameters, including reduced joint pain in osteoarthritis (35–37) and enhanced tendon resilience in athletes (38). Notably, Shilajit supplementation has recently emerged as a novel enhancer of type I collagen synthesis, with a significant rise in serum pro-c1α1 levels after 8 weeks in trained men (39). The clinical effects of collagen appear to be dose- and population-dependent. While 2.5–5 g/day may suffice for skin-related outcomes, higher doses (10–20 g/day) are more effective in muscle recovery, joint health, and Bone mineral density (BMD) enhancement (40–42). Athletes (43, 44) and older adults (32, 45) often exhibit greater responsiveness, likely due to baseline deficits in collagen turnover or repair mechanisms. Despite the promising outcomes, heterogeneity in peptide source, formulation, and outcome measures limits direct comparisons across studies. Long-term trials with standardized endpoints and mechanistic biomarkers (e.g., pro-collagen type I (Pro-C1), C-terminal telopeptide (CTX)) are warranted to validate durability and systemic effects.

### 3.3 Methodological comparison and evidence quality

A comparative evaluation of the clinical trials summarized in Table 2 highlights significant heterogeneity in study design, sample size, dosage, and outcome measures, which collectively influence the strength of evidence. Most studies between 2020 and 2025 were RCTs, with approximately two-thirds adopting double-blind, placebo-controlled designs, while the remainder were open-label or single-arm interventions. This methodological variation affects the internal validity and reproducibility of findings.

Across the 28 clinical trials summarized in Table 2, the average sample size was approximately 93 ± 54 participants (range: 8–300), with most studies enrolling between 60 and 120 subjects. The mean intervention duration was 11.6 weeks, reflecting the predominance of 8- to 12-week RCTs, though a few extended to 18- or 24-week follow-ups. The average daily collagen dosage was 8.4 ± 5.6 g, with dermatologic studies clustering around 2.5–10 g/day and musculoskeletal or metabolic studies using 10–20 g/day. In total, 82% of trials reported at least one significant positive outcome, most commonly improvements in skin hydration and elasticity (44%), joint pain reduction or mobility (29%), and muscle recovery or composition (18%). Only 7% of studies found no measurable difference versus placebo. Collectively, these values underscore a dose- and duration-dependent trend, with higher daily intake and longer interventions yielding more robust and systemic benefits.

Outcome assessment methods varied across research domains. Skin studies employed instrumental techniques (corneometer, cutometer, ultrasound) and standardized photographic grading, while musculoskeletal studies used patient-reported pain scales, strength tests, and biochemical markers such as PINP and CTX. Analytical diversity—ELISA vs. LC–MS biomarker quantification—introduces inter-study variability. Few trials reported compliance monitoring, dietary control, or long-term follow-up beyond 6 months, limiting conclusions about durability and safety.

Overall, while most RCTs demonstrated positive and statistically significant effects, evidence quality is constrained by short intervention periods, lack of standardized collagen formulations, and small sample sizes in specific subpopulations. Future research should emphasize multi-center, longer-term, double-blind RCTs with unified dosage ranges, validated outcome measures, and mechanistic biomarker endpoints to enhance comparability and clinical relevance.

## 4 Collagen supplementation

Collagen supplements vary by source and formulation, each with distinct clinical applications.

### 4.1 Types of collagen supplements

Animal-derived CPs, including marine, bovine, and porcine sources, dominate current use due to their favorable bioavailability and documented efficacy in dermatologic and musculoskeletal conditions. Synthetic analogs and human recombinant collagen are also being explored for regenerative medicine and wound healing, often in conjunction with nanomaterials that mimic native collagen structure and demonstrate hemostatic potential (46) (Table 3).

**Table 3:** Comparison of collagen supplementation types.

Type	Bovine	Marine	Porcine	Chicken
Source	Cows hide and bones	Fish skin and scales	Pig skin and bones	Chicken cartilage
Collagen type	I and III	I	I and III	II
Biological effects	Skin elasticity, joint and bone health	Skin and wound healing	Skin, hair, nails, joint health	Joint and cartilage repair
Bioavailabiltiy	Moderate	High	Moderate	High

An increasing number in clinical research highlights the benefits of CPs. For example, a 12-week randomized trial showed that a supplement containing fish CPs, taurine, cysteine, methionine, iron, and selenium significantly improved hair growth outcomes in participants with alopecia, especially when combined with standard pharmacotherapy (33). Similarly, a 90-day intervention with fish cartilage CPs improved wrinkle appearance and collagen morphology, confirmed by confocal microscopy (47).

In joint health, CPs type II (HC-II) supplementation has been shown to reduce pain and improve musculoskeletal function. A study comparing hydrolyzed collagen type II (HC-II) and an essence of chicken-CP-II blend (EC-HC-II) found significant improvements in grip strength and fat-free mass, alongside reduced joint pain (35). The JUMPFOOD study, currently ongoing (NCT05407194), examines the synergistic effects of CP and vitamin C with exercise therapy in athletes with patellar tendinopathy (48). Moreover, a multi-collagen (types I, II, III) supplement improved pain, balance, and walking ability in patients with osteoarthritis after 8 weeks (35).

### 4.2 Hydrolysed collagen supplements

Native collagen is a large, triple-helical protein that requires enzymatic breakdown for absorption, limiting its bioavailability (49). In contrast, CPs is processed into low molecular weight peptides (3–6 kDa) (50), allowing for more efficient gastrointestinal absorption and systemic distribution (51, 52). Research has demonstrated that CPs, when ingested, are absorbed in the small intestine and can enter the bloodstream as bioactive oligopeptides, which may then exert beneficial effects on collagen metabolism (53, 54). This absorption mechanism highlights the potential for CPs from animal sources to be utilized effectively in dietary supplements, enhancing collagen levels in the body. This inefficiency can hinder the potential therapeutic effects of natural collagen, particularly in conditions like osteoarthritis and skin aging, where increased collagen synthesis is desired (55).

CPs significantly elevate plasma levels of amino acids like proline and hydroxyproline, which are essential for collagen synthesis (56). These peptides stimulate fibroblast and chondrocyte activity (55, 57), enhance hyaluronic acid production, and upregulate collagen type II synthesis (57). This improved efficacy positions CPs as the preferred form for therapeutic use in dermatology and joint health.

### 4.3 Plant-based claims of collagen boosters: evidence and limitations

In recent years, there has been a growing trend toward vegan and plant-based nutrition, a movement that has also extended into the collagen supplement market. While traditional collagen supplements are animal-derived, plant-based alternatives are increasingly marketed as “collagen boosters” to appeal to vegan consumers (58, 59). However, the scientific evidence for collagen supplementation itself remains mixed. While several studies report improvements in skin elasticity and joint function with collagen supplementation (60), these findings are not universally consistent. Many trials have small sample sizes, short durations, or industry sponsorship, which raises concerns about generalizability and potential bias. Furthermore, some reported benefits may be attributable to overall protein intake rather than collagen-specific effects. Plant-based supplements face even greater challenges, as plants do not contain collagen. Claims that bamboo extracts, polysaccharides, or phytoestrogens stimulate fibroblast collagen production are largely unsupported (61–63), with most evidence limited to *in vitro* studies that lack clinical translation. Likewise, antioxidants such as green tea catechins, lycopene, and other polyphenols may reduce oxidative stress and protect existing collagen from degradation (64, 65), but there is no convincing evidence that they promote collagen synthesis or meaningfully improve skin or joint health. These limitations underscore the need for more rigorous evaluation, as the current literature tends to overstate preliminary findings. Emerging technologies may help bridge this gap: biosensors capable of real-time monitoring of collagen-related biomarkers (e.g., hydroxyproline, MMP activity) could improve the objectivity of efficacy assessments, while advanced drug delivery systems may enhance the bioavailability of collagen peptides. Together, these approaches could provide the robust evidence required to distinguish genuine biological effects from marketing exaggeration.

## 5 Electrochemical sensors and collagen delivery systems

Collagen has emerged as a versatile biopolymer carrier for drug delivery, capitalizing on its biocompatibility, biodegradability, and intrinsic tissue affinity. Its native triple-helical structure, enriched with amino acids such as glycine, proline, and hydroxyproline, provides abundant functional groups (–NH₂, –COOH, –OH) for chemical modification, cross-linking, or drug conjugation, thereby enabling both controlled release and targeted delivery. Depending on the formulation strategy, collagen can function as a hydrogel matrix, nanocarrier, or stimuli-responsive scaffold, allowing sustained and localized release of therapeutic agents.

Recent advancements have also integrated collagen with electrochemical biosensors and nanostructured delivery systems (e.g., nanoparticles, hydrogels, microneedle arrays) to achieve precise, real-time monitoring of drug diffusion and targeted release of collagen peptides for optimized therapeutic outcomes. These systems combine collagen’s biological compatibility with the sensitivity and selectivity of sensor platforms, offering a synergistic approach to personalized medicine and dermatologic therapy.

### 5.1 Collagen sensing mechanisms

Electrochemical sensors were chosen for these applications due to their unique advantages over traditional analytical techniques such as ELISA, LC–MS, or spectroscopy. These sensors offer high sensitivity and selectivity, rapid response times, and the ability to perform real-time, *in situ* measurements with minimal sample preparation (8, 66). Such capabilities are particularly valuable in monitoring dynamic processes, such as the controlled release of collagen peptides from delivery matrices, where temporal resolution is critical. Additionally, electrochemical sensors are cost-effective, portable, and amenable to miniaturization, allowing integration with wearable or implantable delivery systems (8). This combination enables continuous monitoring of collagen peptide levels or other biomarkers, which is difficult to achieve with conventional endpoint assays.

Electrochemical collagen sensors rely on the specific recognition of collagen or collagen-derived peptides, followed by conversion of this biochemical interaction into an electrical signal. Common recognition strategies include enzyme-assisted cleavage, antibody or aptamer binding, and molecular imprinting (67, 68). For example, collagenase-sensitive electrodes detect changes in redox-active species upon peptide cleavage (69), while immunosensors use immobilized anti-collagen antibodies to selectively capture collagen molecules, generating measurable changes in current or impedance (70).

Key performance parameters in sensor studies include the limit of detection (LOD), limit of quantification (LOQ), linear range, and selectivity/interference studies (71). These are often determined using spiked biological samples such as serum, plasma, or tissue extracts to mimic real-world applications. For instance, the MIP electrochemical biosensor for serum CTx-I used interdigital capacitive electrodes combined with electrochemical impedance spectroscopy. It exhibited a linear range of 0.1–2.5 ng/mL, a LOD of 0.09 ng/mL, and was validated on sheep serum samples with good correlation to ELISA (12).

Wearable collagen sensors represent a significant advancement, integrating biocompatible collagen matrices with flexible substrates to allow real-time monitoring of collagen dynamics in sweat or interstitial fluid. These systems combine collagen’s natural affinity for extracellular matrix components with sensitive electrochemical transducers, enabling localized and continuous detection for dermatologic and regenerative medicine applications (9, 72, 73).

Overall, incorporating detailed sensing mechanisms and reporting analytical parameters such as LOD, LOQ, linear range, and real sample validation strengthens the understanding of collagen sensor performance and their translational potential in both clinical and consumer applications.

### 5.2 Biosensing platforms for collagen and related biomarkers

In particular, electrochemical sensors based on MIP have shown high promise for detecting and delivering collagen-related biomolecules. MIP are synthetic receptors that replicate the binding sites of target molecules. Electrochemical sensors based on MIP are typically constructed through non-covalent interactions such as hydrogen bonding, electrostatic attraction, and hydrophobic forces between the template and monomers (8, 74). Collagen, being amphiphilic, provides both hydrophilic (–OH, –COOH, –NH₂) and hydrophobic (–CH₂–, –CH₃) groups that can participate in these interactions, enabling the formation of stable and selective imprinted cavities domains (75).

Although electrochemical sensors based on MIP have shown considerable promise for detecting collagen-related biomarkers, most current applications focus on indirect targets such as the CTX-I (12), MMP-1, MMP-8 (76), or hydroxyproline—key indicators of collagen degradation and tissue remodeling (77). For instance, MIP-based impedance sensors have been developed for CTX-I quantification in bone turnover monitoring (12), and graphene oxide–MIP interfaces have been used to detect MMP-8 activity in periodontal disease (76). However, few MIP platforms directly recognize collagen peptides or the intact collagen molecule, largely due to the polymerization challenges posed by its large size, conformational complexity, and limited solubility. Therefore, there remains a critical need to design next-generation MIP sensors capable of selectively imprinting collagen fragments or characteristic peptide motifs, which would enable direct, real-time assessment of collagen supplementation, metabolism, and therapeutic response in biomedical and cosmetic contexts.

Consequently, collagen-based MIP can serve dual purposes: (1) as recognition elements in biosensors for detecting biomarkers or drug concentrations, and (2) as functional components of delivery systems, enabling feedback-controlled release in response to physiological cues such as pH, temperature, or enzymatic activity. This convergence of biopolymer chemistry, nanotechnology, and electrochemical sensing represents a key frontier in next-generation collagen therapeutics, particularly for skin regeneration, anti-aging formulations, and musculoskeletal repair.

Beyond electrochemical transduction, optical and piezoelectric platforms have also been applied to study collagen or its degradation products.

For example, surface plasmon resonance (SPR) biosensors allow label-free detection of MMP activity and collagen–ligand interactions (76, 78), while fluorescence-based assays using collagen-binding peptides (CBPs) enable visualization of matrix remodeling (79). Quartz crystal microbalance (QCM) sensors have also been used to monitor collagen film deposition and cross-linking kinetics (80).

### 5.3 Collagen-based delivery systems

Whole collagen molecules are difficult to use as biological drugs due to their insolubility, but collagen fragments of lower molecular weight, resulting from enzymatic degradation, are more suitable for drug development and have gained attention in pharmacological research. These collagen-derived fragments, known as matrikines or matricryptins, exhibit diverse biological activities, with some proving beneficial as potential therapeutic agents, while others may have harmful effects on health (46). Scientists proposed a simple mathematical model to estimate cartilage damage, considering factors such as age, obesity, gender, and overloading, while accounting for the cartilage’s self-repair capacity. The model assumes homogeneous distribution of cartilage components, including collagens, proteoglycans, and glycoproteins, and uses Python’s Numpy library for modeling and predictions. The results indicate that cartilage damage increases by 6.5% for each unit increase in BMI above 27, with critical damage occurring between the ages of 44 and 64, which aligns with clinical data (81).

Despite the ability to predict cartilage damage using mathematical models, electrochemical methods and sensors offer a real-time monitoring of collagen distribution and structural changes within the tissue. For example, an electrically active composite material made from a biocompatible hydrogel, gelatin methacryloyl (GelMA), and a conducting polymer, poly (3,4-ethylenedioxythiophene), is a conducting polymer hydrogel for controlled protein delivery. The short-term release of a model protein was controlled over 4 h with electrical stimuli, and extended-release studies over 21 days demonstrated a bimodal release profile influenced by GelMA degradation and electrical stimulation. This electroactive, cytocompatible material is ideal for tissue engineering applications that require targeted, spatio-temporal delivery of therapeutic proteins (82).

Peripheral nerve repair faces challenges due to the limited regenerative capacity of nerves and complications with autografts. Axons and myelin sheaths are encased by the endoneurium, which contains aligned collagen fibers, while the perineurium, composed of flattened fibroblasts and collagen, bundles the axons into fascicles (83). The fibrocollagenous epineurium surrounds the fascicles and vasculature (arteries and veins), holding them together to form the nerve trunk. Electrical stimulation has shown potential to enhance nerve regeneration by modulating the bioelectrical environment and promoting a reparative immune response. Electrically conductive polymer - based biomaterials offer advantages for nerve repair, combining flexibility, ionic-electronic conductivity, biocompatibility, and ease of fabrication (83).

Electrochemical therapy has also been explored to modulate dermal collagen architecture for scar treatment. Pham et al. described the use of electrochemical therapy creates focal pH gradients in skin, altering dermal collagen for scar treatment. By applying an electrical potential through platinum electrodes in porcine skin, the researchers observed biochemical and structural changes, including collagen denaturation, as confirmed by histology and imaging techniques (84). Another study investigated the use of short-duration topical co-iontophoresis to deliver cationic buflomedil hydrochloride (BUF) and anionic dexamethasone phosphate (DEX-P) to the oral mucosa in a novel concentric experimental setup. The iontophoresis procedure applied a constant current of 3.0 mA for 5, 10, and 20 min, resulting in a significant increase in drug delivery—BUF increased by nearly 6.5-fold and DEX-P by over 5-fold compared to passive controls. Quantification of drug migration revealed substantial lateral ion migration, with higher drug delivery observed in the cathodal and inter-electrode areas. The results suggest that short-duration co-iontophoresis can effectively and safely enhance local bioavailability, offering a promising and patient-friendly treatment for oral diseases like oral submucous fibrosis related to excessive collagen production (85).

### 5.4 From peptide self-assembly to functional biorecognition elements

The development of collagen mimetic peptides (CMPs) has garnered significant interest for their potential applications in tissue engineering, regenerative medicine, and biomaterials. In recent years, the self-assembly of small CMPs into large-scale collagen-like structures has been widely explored, offering new strategies for the creation of innovative biomaterials (86).

A polypyrrole (PPy)-based bipolar electrostimulation (BPES) system was applied for living cells. By utilizing PPy films doped with dextran sulfate and collagen (PPy-DS/collagen), the system demonstrated reversible and recoverable bipolar electrochemical activity under low DC voltage (<5.5 V). The BPES prototype, which uses these materials as bipolar electrodes, was successfully applied to rat pheochromocytoma cells, showing enhanced cell proliferation and differentiation (87).

The use of metal–organic frameworks, specifically collagen encapsulated strontium ranelate@PCN-224 enhanced the osseointegration of zirconium alloy implants by loading osteogenic drugs such as strontium ranelate (SR). The synthesized PCN-224, with a large surface area and specific pore structure, successfully adsorbed SR without altering its crystal structure, exhibiting a maximum adsorption capacity of 112.67 ± 2.14 mg g − 1*. In vitro* experiments demonstrated that the SR-loaded PCN-224 coating, when applied to zirconium alloy, promoted osteoblast proliferation and differentiation, offering a promising strategy to improve bone-implant integration (88). Another study explored the incorporation of wool, choline dihydrogen phosphate ([Ch][DHP]), or choline serinate ([Ch][Ser]) ionic liquids (ILs) into native collagen formulations to create films via compression molding. Scanning electron microscopy confirmed the good dispersion of additives without agglomeration, while differential scanning calorimetry and infrared spectroscopy demonstrated that the collagen fibrillary structure remained intact. X-ray diffraction patterns supported these findings, showing that the addition of wool or ILs enhanced tensile strength, electrical conductivity, and antistatic behavior. The films also exhibited a high dielectric constant, primarily from mobile charge contributions, highlighting the potential of these collagen-based materials for a wide range of sustainable applications (89).

Scientists synthesized the CMP (Pro-Hyp-Gly) 10, referred to as POG10, and investigated its interactions with varying concentrations of choline chloride (ChCl) using a variety of biophysical techniques, including circular dichroism, UV–vis spectroscopy and other. For the first time, scientists demonstrated how ChCl can be used to modulate the stability and aggregation of CMP triple helices, shedding light on the factors that influence the stability of these structures (86).

### 5.5 Collagen-based hydrogels, nanoparticles and liposomal systems

Collagen-based hydrogels and nanoparticles have gained substantial attention as next-generation drug delivery platforms, owing to their tunable mechanical strength, high water content, and intrinsic bioactivity. Collagen’s natural cell-adhesive motifs and enzymatic degradability enable it to serve as a responsive and biocompatible matrix for sustained and localized release of therapeutic agents. These systems can be engineered to respond to biological or external stimuli—such as enzymatic degradation, pH, temperature, or electrical input—allowing for precise spatiotemporal control of drug release (90, 91). Their key advantages include biocompatibility, minimal immunogenicity, and versatility in integrating growth factors, small molecules, or peptides for tissue regeneration and wound healing applications (92).

Several examples already discussed illustrate the diversity of collagen-based hydrogel and nanoparticle systems. For instance, electrically conductive GelMA–PEDOT hydrogels enabled electroresponsive protein release over a 21-day period (82), while collagen-encapsulated metal–organic frameworks such as SrR@PCN-224 enhanced osteoblast differentiation and osseointegration on zirconium alloy implants S (88). These studies highlight the capacity of collagen matrices to function as bioactive carriers capable of synergizing with nanostructured or electroactive components to improve delivery precision and therapeutic outcomes.

Beyond laboratory research, collagen-based nanostructures have also entered translational and commercial stages. For example, Liposovit®-Collagen Direct, incorporates liposomal hydrolyzed fish collagen type I (< 300 nm particle size) to improve bioavailability and dermal absorption, representing one of the first marketed liposomal collagen formulations for skin and joint health. Similarly, injectable collagen–polyethylene glycol hydrogels loaded with stem cell factor have been developed for diabetic wound healing, demonstrating accelerated re-epithelialization and improved vascularization (93). Nanoparticulate mineralized collagen-glycosaminoglycan scaffolds have further shown bone-regeneration potential without exogenous growth factors *in vivo* (94). Commercial collagen wound dressings, such as absorbable collagen matrices, have already gained FDA 510(k) clearance, confirming their biocompatibility and clinical applicability (95).

Despite these achievements, several limitations persist. Collagen hydrogels often suffer from rapid enzymatic degradation and weak mechanical integrity under physiological loading conditions, while nanoparticle formulations may face challenges related to reproducibility, limited drug loading capacity, and undesirable burst release effects. The integration of conductive polymers or inorganic nanoparticles, though beneficial for electrical responsiveness and bioactivity, can raise cytotoxicity concerns if residual monomers or metal ions leach from the matrix. Achieving a stable balance between biodegradability, biofunctionality, and electrochemical responsiveness remains a central materials-engineering challenge (82).

The increasing scientific interest in collagen-based nanotechnology and hydrogel systems reflects both their practical advantages and their appeal for translational medicine. They are relatively simple to synthesize, allow control over drug distribution, and can be monitored for their movement and release dynamics within biological environments. Moreover, the integration of nanotechnology enhances marketing potential—patients and consumers are drawn to innovative formulations that promise faster and more visible effects. Liposomes, in particular, are a widely adopted vehicle in pharmaceutical and cosmetic industries because of their established safety profile and enhanced delivery efficiency. However, while many collagen-containing liposomal and hydrogel formulations are marketed for cosmetic or nutraceutical purposes, only a few have undergone rigorous clinical testing to confirm long-term efficacy or systemic safety.

This disparity underscores a critical translational gap: although numerous studies report promising *in vitro* and *in vivo* performance of collagen-based nano-hydrogel systems, there is still a lack of regulatory-approved, sensor-integrated, or collagen-fragment-specific delivery systems. Bridging this gap requires standardized protocols for evaluating stability, release kinetics, and biocompatibility, as well as integrated analytical methods—such as electrochemical or optical biosensors—to monitor collagen degradation and therapeutic response in real time. Ultimately, advancing these hybrid materials from laboratory prototypes to clinically approved therapies will depend on the successful reconciliation of their physicochemical complexity with practical safety, manufacturability, and regulatory compliance.

## 6 Conclusion

Collagen, the most abundant structural protein in the human body, underpins the integrity and function of skin, cartilage, and bone. Its biosynthesis is a complex, multistep process initiated in the endoplasmic reticulum and catalyzed by key enzymes such as prolyl hydroxylases and peptidyl-prolyl cis-trans isomerases. Dysregulation or mutation in these pathways can impair tissue homeostasis, repair, and extracellular matrix remodeling.

Contemporary collagen research increasingly bridges the domains of nutritional supplementation, biosensing, and drug delivery, forming an integrated framework that supports precision medicine and regenerative healthcare. Collagen supplementation provides essential substrates—hydrolyzed peptides and amino acids—that stimulate endogenous collagen synthesis and promote tissue repair. However, interindividual variability in absorption, metabolism, and physiological response leads to heterogeneous clinical outcomes, underscoring the need for real-time monitoring tools capable of assessing collagen turnover, degradation, and remodeling *in vivo*.

Emerging electrochemical and optical biosensing platforms—including molecularly imprinted polymer (MIP)-based sensors, surface plasmon resonance systems, and fluorescence assays utilizing collagen-binding peptides (CBPs)—enable dynamic, label-free detection of key collagen-related biomarkers such as CTX-I, MMPs, and hydroxyproline. These sensing tools can provide quantitative feedback on collagen metabolism, facilitating personalized supplementation strategies that optimize dose, source, and treatment duration according to patient-specific biomarker profiles.

Concurrently, collagen-based hydrogels, nanoparticles, and electroresponsive scaffolds function as smart delivery systems capable of controlled, stimuli-responsive release of therapeutic agents—including peptides, growth factors, and antioxidants. Integration of biosensors within these delivery matrices could establish closed-loop feedback systems, wherein biomarker fluctuations (e.g., collagen degradation or peptide concentration) directly trigger or modulate therapeutic release in real time. Such convergence of sensing and delivery may significantly enhance treatment precision and reduce systemic side effects.

Moving beyond population-level interventions, personalized collagen therapeutics will rely on integrative strategies that combine mathematical modeling, biosensing, and molecular diagnostics to monitor and guide therapy. Moreover, collagen mimetic peptides (CMPs) provide novel avenues for regenerative medicine, offering bioinspired building blocks that mimic native collagen’s structural and functional properties.

In summary, advancing collagen research requires a systems-level approach that connects biochemical mechanisms, sensor-driven diagnostics, and material-based delivery technologies. This conceptual integration will form the foundation for the next generation of data-informed, adaptive collagen therapies in dermatology, orthopedics, and regenerative healthcare.
